# A High-Relative-Bandwidth Doherty Power Amplifier with Modified Load Modulation Network for Wireless Communications

**DOI:** 10.3390/s23052767

**Published:** 2023-03-02

**Authors:** Haipeng Zhu, Zhiwei Zhang, Chao Gu, Xuefei Xuan

**Affiliations:** 1 School of Electronics and Information, Hangzhou Dianzi University, Hangzhou 310018, China; 2 ECIT Institute, Queen’s University Belfast, Belfast BT3 9DT, UK

**Keywords:** broadband, Doherty power amplifier, load modulation network, relative bandwidth

## Abstract

This paper presents a novel load modulation network to realize a broadband Doherty power amplifier (DPA). The proposed load modulation network consists of two generalized transmission lines and a modified coupler. A comprehensive theoretical analysis is carried out to explain the operation principles of the proposed DPA. The analysis of the normalized frequency bandwidth characteristic shows that a theoretical relative bandwidth of approximately 86% can be obtained across a normalized frequency range of 0.4–1.0. The complete design process that allows the design of the large-relative-bandwidth DPA based on derived parameter solutions is presented. A broadband DPA operating between 1.0 GHz and 2.5 GHz was fabricated for validation. Measurements demonstrate that the DPA can deliver an output power of 43.9–44.5 dBm with a drain efficiency of 63.7–71.6% in the 1.0–2.5 GHz frequency band at the saturation level. Moreover, a drain efficiency of 45.2–53.7% can be obtained at the 6 dB power back-off level.

## 1. Introduction

The rapid emergence of wireless communication applications that need to transmit large amounts of data such as wireless sensing networks has encouraged the development of RF front-end technology that can support high peak-to-average ratio (PAPR) signals with large bandwidth [1]. Power amplifiers (PAs) are required in modern wireless communication application systems to handle broad bandwidth (BW) and high PAPR simultaneously for the purpose of increasing data transmission rates. Traditional single-device PAs, such as class-F [2], class-E [3], and class-J [4], cannot meet the requirements of efficiently amplifying signals with high PAPR. Load modulation PAs, such as Doherty [5], out-phasing [6], and load modulation balance amplifiers (LMBAs) [7], can maintain high efficiency at the power back-off (OBO) level and are promising candidates for meeting the stringent linearity requirements. Currently, DPAs are widely used in wireless communications due to their easy-to-implement architecture [8,9]. However, the bandwidth of traditional Doherty PAs is restricted by impedance conversion networks, offset lines, matching networks, and package parameters of transistors [10,11,12,13]. Therefore, DPAs can only maintain the ideal performance at the OBO level in a narrow band. This bandwidth limitation greatly hinders the use of DPAs in RF transceivers that require high data BW and high PAPR.

Recently, some methods have been proposed to improve the bandwidth of Doherty PAs [14,15,16,17,18,19,20,21,22,23,24]. The finite impedance of the auxiliary PA branch at the OBO level is employed to enhance the OBO performance in a large bandwidth [14,15]. In addition, the introduction of complex load impedances to achieve better impedance conditions in a large bandwidth has been validated [16,17]. Post-matching techniques can reduce the impedance transformation ratio of output matching networks of main PA and auxiliary PA branches so as to provide larger bandwidth [18,19,20,21]. In addition, some continuous-mode PAs have been developed to have the ability to increase the impedance design space, thus widening the bandwidth of DPAs [22,23,24]. These technologies can ameliorate the impedance-matching condition of DPAs to expand the bandwidth of DPAs to a certain extent. However, due to the inherent limitations of a DPA’s load modulation network (LMN) structure, impedance mismatch at the OBO level is still inevitable. This will reduce the efficiency of the DPAs at the OBO level. Therefore, it is necessary to study a new LMN structure in order to realize more accurate impedance conversion at the OBO level in a large bandwidth while keeping Doherty load modulation behaviors.

A lot of effort has been made to realize larger bandwidth by altering the LMN structure of DPAs [25,26,27,28]. Recently, especially in [29,30], novel LMN structures have been proposed to extend the bandwidth, where the precise impedance can be realized using specific analysis at the OBO level. However, the relative bandwidth is still limited since both these analyses are based on traditional transmission lines, resulting in higher normalized frequency bands, such as 0.8–1.2 in [29] and 0.7–1.3 in [30].

In this paper, we propose a novel LMN structure including a modified coupler and two generalized transmission lines. The modified coupler is used in an LMN for the first time to achieve the required impedance such that the combination of the coupler and generalized transmission lines leads to an almost constant impedance at the OBO level in the lower normalized frequency band of 0.4–1, which means an ideal efficiency can be obtained in a larger relative bandwidth compared with previous works, e.g., [29,30]. A theoretical 86% relative bandwidth can be obtained when the proposed LMN is utilized. Furthermore, a more direct and straightforward analysis based on the normalized frequency and impedance condition immediately follows, from which the corresponding design parameters follow. This analysis allows the proposed LMN to be designed for practical situations, including different working frequencies and OBO levels. Compared with the previous broadband DPAs, the proposed DPA significantly expands the relative bandwidth. The structure of this paper is as follows: The theory of the proposed LMN is shown and the corresponding design equations are given in Section 2. Section 3 presents the complete design process, and simulations are given in detail. In Section 4, experiments are shown to validate the proposed DPA. The conclusions of this work are given in Section 5.

## 2. Analysis of DPA Theories

**Figure 1:**
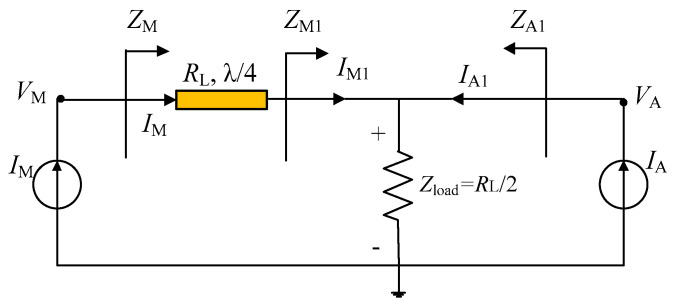
Conventional DPA topology.

The conventional DPA topology is shown in Figure 1; here, the transistor is equivalent to an ideal current source. The conventional DPA has a main PA branch and an auxiliary PA branch, with a load of *R*_L_/2 at the combiner. In addition, a quarter-wavelength transmission line is used in the main PA branch in order to achieve impedance conversion [5]. According to the DPA theory [5], several impedance relationships are given as

(1) $$Z_{M1,\mathrm{SAT}}=2\cdot Z_{\mathrm{load}}$$

(2) $$Z_{M1,\mathrm{OBO}}=Z_{\mathrm{load}}$$

(3) $$Z_{A1,\mathrm{SAT}}=2\cdot Z_{\mathrm{load}}$$

(4) $$Z_{A1,\mathrm{OBO}}=\infty$$

where *Z*_M1_ and *Z*_A1_ are the load impedances of the main and auxiliary PA branches, respectively. *Z*_M_ and *Z*_A_ represent the load impedances of devices for main and auxiliary PAs, respectively. The subscripts SAT and OBO refer to impedance conditions at the saturation and OBO levels, respectively. In the traditional DPA topology, the λ/4 line is responsible for converting the impedance well only at the center frequency, thus restricting the bandwidth of the resulting DPAs.

**Figure 2:**
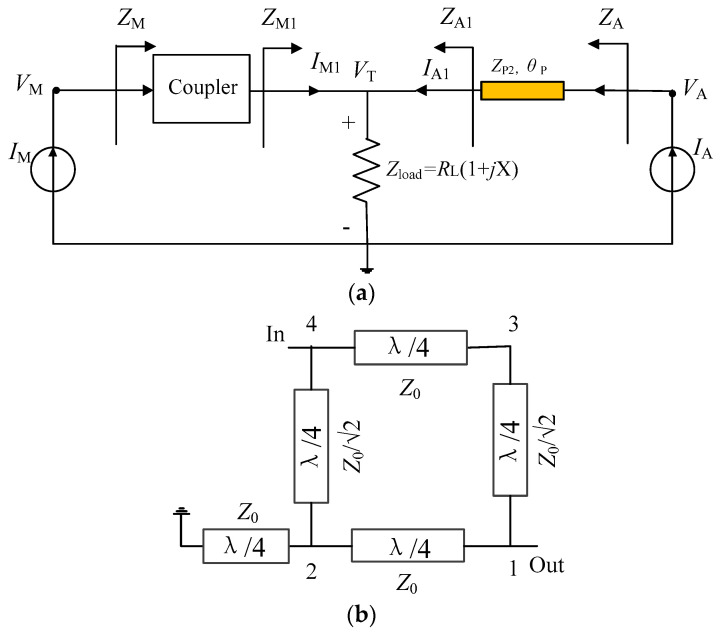
(**a**) Proposed DPA topology; (**b**) modified coupler.

In this work, a modified load modulation network is proposed to address this issue, as shown in Figure 2a. In this scheme, the modified coupler is used to realize the impedance conversion at the main PA branch. The DPA load impedance is set to a complex value *R*_L_ (1 + *j*X). The auxiliary PA branch also includes a generalized transmission line to represent the output network that is employed to obtain finite impedance *Z*_A1,OBO_ (unlike that of infinity in traditional DPAs). The modified coupler structure shown in Figure 2b consists of a traditional branch line coupler and a reactance connected to port 2. Port 4 and port 1 are input and output terminals, respectively, while port 3 is open-circuited. The relationship between the four ports for this coupler arrangement is expressed as

(5) $$\left[\begin{array}{c} V_{1}\\ V_{2}\\ V_{3}\\ V_{4} \end{array}\right]=Z_{0}\left[\begin{array}{cccc} 0 & +j & -j\sqrt{2} & 0\\ +j & 0 & 0 & -j\sqrt{2}\\ -j\sqrt{2} & 0 & 0 & +j\\ 0 & -j\sqrt{2} & +j & 0 \end{array}\right]\left[\begin{array}{c} I_{1}\\ I_{2}\\ I_{3}\\ I_{4} \end{array}\right]$$

where *V* and *I* refer to the voltage and current of ports of the coupler. Subscripts 1, 2, 3, and 4 represent the port numbers. *Z*_0_ is the system impedance. Based on the condition of terminals shown in Figure 2b, the following relationships are obtained:

(6) $$Z_{2}=jZ_{0}\tan\beta l=jZ_{0}\tan\frac{\pi }{2}f$$

*I*_3_ = 0 (7)

where *β* is the propagation constant. *l* is the physical length of the transmission line. *Z*_2_ represents the impedance of port 2.

As *f* is the normalized frequency, the relationship between the impedance of port 4 *Z*_4_ and the impedance of port 1 *Z*_1_ is derived as

(8) $$Z_{4}/Z_{1}=\left(2+jtan\frac{\pi }{2}f\right)$$

As expressed in (8), *Z*_4_/*Z*_1_ has a constant resistance, as required for DPA synthesis [23]. Moreover, the resistance is independent of the normalized frequency *f*, which provides the possibility to realize operation in a lower normalized frequency band than the previous works [29,30]. Next, an analysis of this LMN and corresponding parameter solutions are given.

### 2.1. Power Back-Off Level

**Figure 3:**
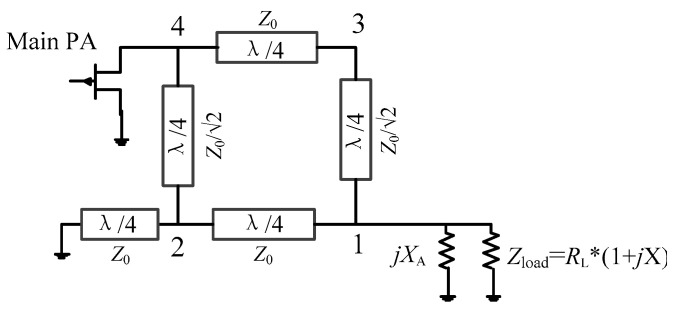
Equivalent schematic diagram of the main PA branch at the OBO level.

Figure 3 shows the equivalent schematic diagram of the main PA branch at the power back-off level. As shown in Figure 3, the load impedance of the main PA branch *Z*_M1,OBO_ (*Z*_1_) is as follows:

(9) $$Z_{M1,\mathrm{OBO}}=Z_{1}=\frac{\left(1+jX\right)\left(jX_{A}\right)}{1+jX+jX_{A}}=\frac{\left(jX_{A}-XX_{A}\right)}{1+j\left(X+X_{A}\right)}R_{L}$$

where *X*_A_ refers to the normalized reactance of the auxiliary PA branch at the OBO level.

After substituting (9) to (8), the load impedance of the main PA transistor *Z*_M,OBO_ (*Z*_4_) can be obtained as

(10) $$Z_{M,\mathrm{OBO}}/R_{\mathrm{OPT}}=Z_{4}/R_{\mathrm{OPT}}=\left(1+j0.5tan\frac{\pi }{2}f\right)\frac{\left(jX_{A}-XX_{A}\right)}{1+j\left(X+X_{A}\right)}$$

where *R*_L_ = 0.5*R*_OPT_.

In order to realize an OBO of 6 dB, the real part of *Z*_4_ $Re(Z_{4}/R_{\mathrm{OPT}})$ should be set equal to 2. Then, we can deduce the following two expressions about *X*_A_:

(11) $$X_{A}=\frac{-\left(tan\frac{\pi }{2}f+X^{2}tan\frac{\pi }{2}f+4X\right)+\sqrt{{\left(tan\frac{\pi }{2}f+X^{2}tan\frac{\pi }{2}f+4X\right)}^{2}-4*\left(Xtan\frac{\pi }{2}f\right)\left(2+2X^{2}\right)}}{4\left(Xtan\frac{\pi }{2}f\right)}$$

(12) $$X_{A}=\frac{-\left(tan\frac{\pi }{2}f+X^{2}tan\frac{\pi }{2}f+4X\right)-\sqrt{{\left(tan\frac{\pi }{2}f+X^{2}tan\frac{\pi }{2}f+4X\right)}^{2}-4*\left(Xtan\frac{\pi }{2}f\right)\left(2+2X^{2}\right)}}{4\left(Xtan\frac{\pi }{2}f\right)}$$

**Figure 4:**
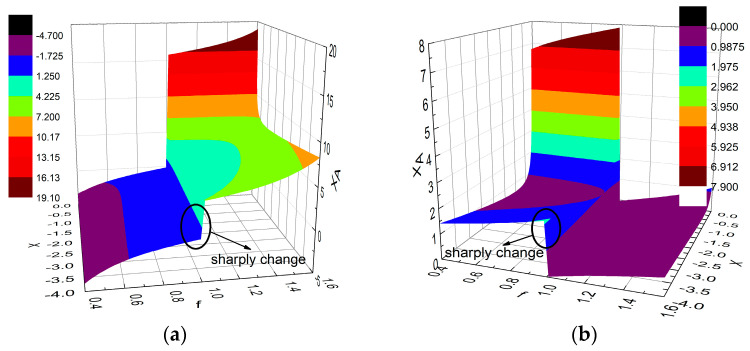
Relationships between *X*_A_, *X*, and *f*: (**a**) relationship expressed by (11); (**b**) relationship expressed by (12).

Figure 4a,b display the relationships expressed by (11) and (12), respectively. Based on Figure 4, we can now choose the appropriate *X*_A_ and *X* to realize the desired OBO, in this case, 6 dB, for different values of *f*. Indeed, we can let $Re(Z_{4}/R_{\mathrm{OPT}})$ be equal to a different value for obtaining different OBO values if desired. For example, an OBO of 9 dB can be obtained by setting $Re(Z_{4}/R_{\mathrm{OPT}})$ = 4.

As shown in Figure 4, there are two solutions for *X* and *X*_A_ in the normalized frequency band of 0.4–1.6. This provides more freedom to satisfy the impedance requirements of DPAs at the specified saturation level. Moreover, the value of *X*_A_ changes sharply around *f* = 1. This variation hinders the realization of ultra-wideband DPAs in the normalized frequency range of 0.4–1.6. Therefore, the proposed topology can theoretically only achieve wideband DPA in the 0.4–1.0 or 1.0–1.6 frequency range. In order to obtain the maximum relative bandwidth, this paper chooses the frequency range of 0.4–1.0 for the following analysis and design.

### 2.2. Saturation Level

**Figure 5:**
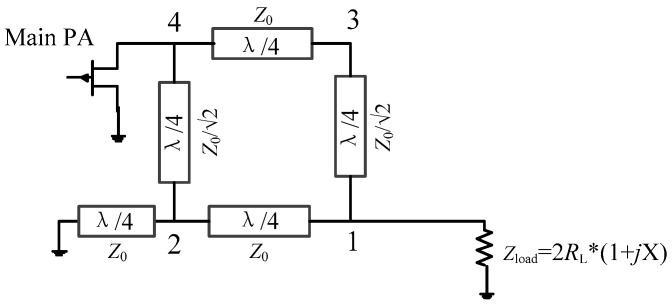
Equivalent schematic diagram of the main PA branch at the saturation level.

Figure 5 shows the equivalent schematic diagram of the main PA branch at the saturation level. The load impedance of the main PA branch is 2*R*_L_(1 + *jX*). According to the DPA theory, the load impedance of the main PA transistor *Z*_M,SAT_(*Z*_4_) can be expressed as

(13) $$Z_{M,\mathrm{SAT}}/R_{\mathrm{OPT}}=Z_{4}/R_{\mathrm{OPT}}=\left(2+jtan\frac{\pi }{2}f\right)\left(1+jX\right)$$

At the saturation level, the real part of *Z*_4_ $Re(Z_{4}/R_{\mathrm{OPT}})$ should be equal to 1. Hence, the parameter *X* can be determined as

(14) $$X=\left(\frac{1}{tan\frac{\pi }{2}f}\right)$$

The values of parameter *X* at several representative frequencies are listed in Table 1.

Then, by substituting (14) to (11) and (12), the values of *X*_A_ at these frequencies *f* can be further obtained as shown in Table 1. We note here that *X*_A_ has two solutions caused by (11) and (12), while *X* only has one solution.

Once we obtain the values of these parameters, as listed in Table 1, we can further derive the theoretical drain efficiency characteristic of the proposed DPA. The theoretical drain efficiency of the proposed DPA at the desired OBO level can be derived as follows.

If |*Z*_M, OBO_| ≤ 2*R*_OPT_, the main PA does not reach the saturation level; thus, the current *I*_M, OBO_ is *I*_max_/4, and the voltage *V*_M, OBO_ is equal to *I*_M, OBO_*Z*_M, OBO_. The output power of the DPA at the OBO level can be calculated as follows:

(15) $$P_{\mathrm{out},\mathrm{OBO}}=0.5\times Re\left(V_{M,\mathrm{OBO}}^{*}\cdot I_{C,\mathrm{OBO}}\right)=\frac{1}{32}I_{\max}^{2}Re\left(Z_{M,\mathrm{OBO}}\right)$$

If |*Z*_M, OBO_| is larger than 2*R*_OPT_, the main PA is overdriven. The output power of the DPA at the OBO level should be calculated as

(16) $$P_{\mathrm{out},\mathrm{OBO}}=0.5\times Re\left(V_{M,\mathrm{OBO}}^{*}\cdot I_{M,\mathrm{OBO}}\right)=\frac{1}{2}\frac{V_{\mathrm{dc}}^{2}}{\left|Z_{M,\mathrm{OBO}}\right|}\cdot \frac{Re\left(Z_{M,\mathrm{OBO}}\right)}{\left|Z_{M,\mathrm{OBO}}\right|}$$

The DC power consumption of the DPA can be assumed as a constant level [17], which is expressed as

(17) $$P_{\mathrm{dc}}=V_{\mathrm{dc}}I_{\mathrm{dc}}=\frac{V_{\mathrm{dc}}I_{\max}}{2\pi }$$

Then, the drain efficiency (DE) at the OBO level is derived as

(18) $$DE_{\mathrm{OBO}}=\frac{\pi }{4}\frac{Re\left(Z_{M,\mathrm{OBO}}\right)}{2R_{\mathrm{OPT}}},\;\;\;\;\left|Z_{M,\mathrm{OBO}}\right|\leq 2R_{\mathrm{OPT}}$$

(19) $$DE_{\mathrm{OBO}}=\frac{\pi }{4}\frac{2R_{\mathrm{OPT}}}{\left|Z_{M,\mathrm{OBO}}\right|}\frac{Re\left(Z_{M,\mathrm{OBO}}\right)}{\left|Z_{M,\mathrm{OBO}}\right|},\;\;\;\;\left|Z_{M,\mathrm{OBO}}\right|>2R_{\mathrm{OPT}}$$

**Figure 6:**
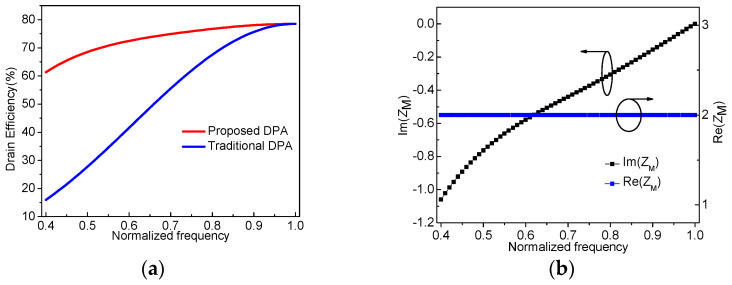
Drain efficiency and impedance versus the normalized frequency: (**a**) drain efficiency versus the normalized frequency; (**b**) impedance *Z*_M_ versus the normalized frequency.

Based on the above values of *X* and *X*_A_ listed in Table 1 and (18) and (19), the drain efficiency at the OBO level can be calculated, as shown in Figure 6a. In addition, for comparison, the drain efficiency for a traditional DPA is added in Figure 6a. It is obvious that the proposed DPA can maintain a higher drain efficiency over a wide bandwidth. In order to ensure a stable power output, we define the real part of the impedance *Z*_M,OBO_ as 2*R*_OPT_ without specifying the imaginary part. Therefore, the drain efficiency of the proposed DPA reduces with frequency, caused by the undesired imaginary part of *Z*_M,OBO_. Figure 6b displays the frequency characteristic of *Z*_M,OBO_, which indicates an imaginary part that varies with normalized frequency. Moreover, the higher *X*_A_, the higher the undesired imaginary part that is obtained based on (10). It is seen from Table 1 that the first solution of *X*_A_ (*X*_A1_) has a smaller value than the second solution *X*_A2_. Therefore, the solution for *X*_A1_ in Table 1 is taken in the following analysis.

Next, we use a simple method to reduce the effect of this variable imaginary part on efficiency.

### 2.3. Improved Main PA Branch

**Figure 7:**
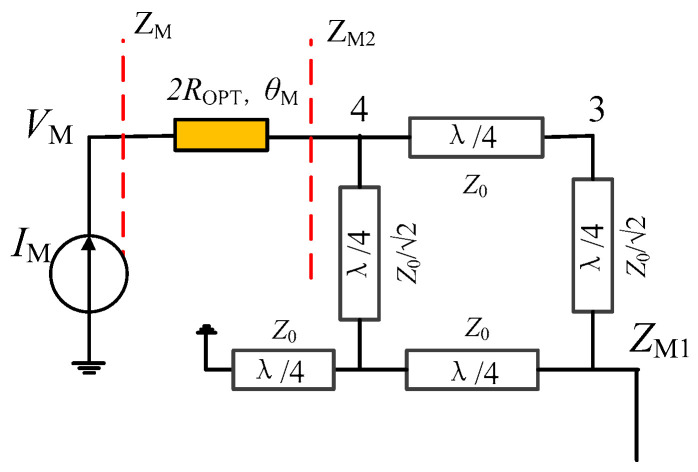
Improved main PA branch.

As shown in Figure 6a, the drain efficiency of the proposed DPA is better than that of the traditional DPA. However, the drain efficiency can still drop to about 20% from the ideal drain efficiency. We now propose an improved main PA branch to further slow down the tendency of drain efficiency to decrease with normalized frequency. Figure 7 shows the topology of the improved main PA branch. Here a transmission line is inserted between the coupler and the drain of the transistor. Therefore, the impedance relationship between impedance *Z*_M_ and *Z*_M2_ can be expressed as

(20) $$Z_{M}=2R_{\mathrm{OPT}}\frac{Z_{M2}+j2R_{\mathrm{OPT}}\tan\theta_{M}}{2R_{\mathrm{OPT}}+jZ_{M2}\tan\theta_{M}}$$

where $\theta_{M}=artan\left(Im\left(Z_{M2}\right)/2\right)$.

**Figure 8:**
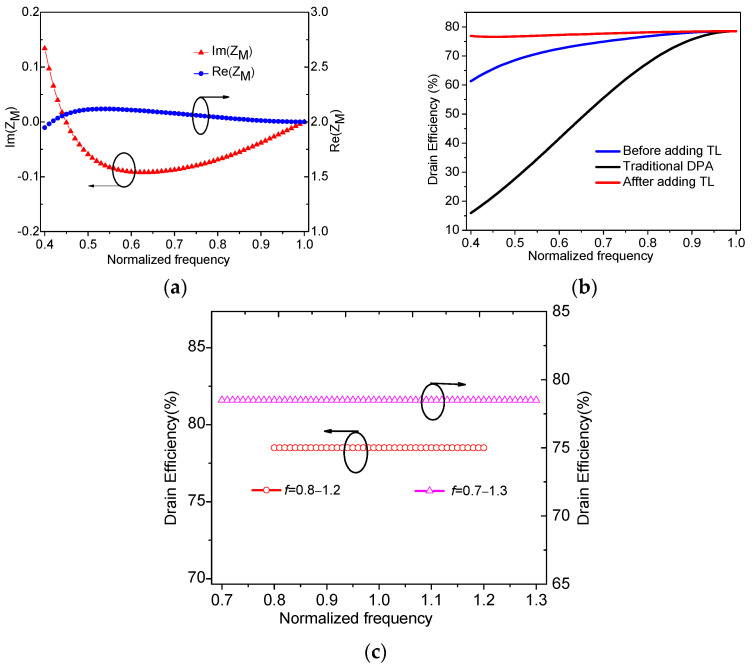
Frequency characteristics: (**a**) impedance *Z*_M,OBO_; (**b**) theoretical drain efficiency in this work; (**c**) theoretical drain efficiency of DPAs in [29,30].

Then, we can obtain the frequency characteristics of impedance *Z*_M,OBO_ as shown in Figure 8a. Comparing Figure 8a with Figure 6b, it can be seen that the imaginary part of *Z*_M,OBO_ in Figure 8a lies closer to zero. In addition, the corresponding drain efficiency at the OBO level is calculated in Figure 8b by using (18) and (19). As shown in Figure 8b, the drain efficiency of the improved main PA roughly maintains a constant profile across the normalized frequency band of 0.4–1. In addition, Figure 8c shows the drain efficiency of DPAs in [29,30], where the normalized frequency bands are 0.8–1.2, and 0.7–1.3 for [29,30], respectively. As shown in Figure 8b,c, the presented DPA has the ability to work in a lower normalized frequency band so as to have a larger relative bandwidth.

**Figure 9:**
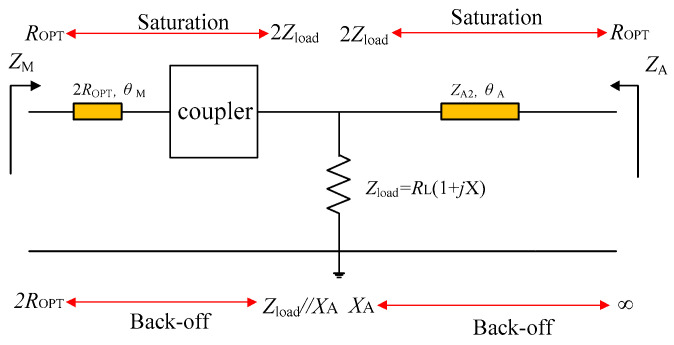
Impedance transformation of the proposed DPA.

Based on the above analysis, the impedance transformation of the proposed DPA is displayed in Figure 9. As shown in Figure 9, at the saturation level, the load impedances of the main PA branch and the auxiliary PA branch are both *Z*_load_, and their values are complex. At the same time, the load impedances of the two PAs’ transistors are both *R*_OPT_. The impedance conversion situation is the same as that of a conventional DPA, except that the load impedance *Z*_load_ of DPA is a complex impedance. At the OBO level, regarding the auxiliary PA branch, the impedance of this branch is finite and is defined as *jX*_A,_ while the load impedance of the device is infinite. This impedance conversion is realized by employing the generalized transmission line. At the OBO level, regarding the main PA branch, the impedance of this branch is *Z*_load_ in parallel with *jX*_A_, which is different from that of the traditional DPA due to the introduction of the finite impedance of the auxiliary PA branch, while the load impedance of the transistor is 2*R*_OPT_. This impedance conversion as required by the main PA is achieved by employing the modified coupler and the injected transmission line. The utilization of this modified coupler and the injected transmission line allows DPA operation with an acceptable efficiency into a lower normalized frequency band so as to achieve a larger relative bandwidth as compared to previous DPAs in the literature. Furthermore, the introduction of the finite load impedance of the auxiliary PA branch and the complex load impedance of the DPA effectively assists in meeting the impedance requirements required for the modified coupler deployment.

The parameters of the generalized transmission line in the auxiliary PA branch are easily obtained by using the following relationship:

(21) $$Z_{A}=Z_{A2}\frac{Z_{A1}+jZ_{A2}\tan\theta_{A}}{Z_{A2}+jZ_{A1}\tan\theta_{A}}$$

where *Z*_A1_ is equal to *jX*_A_ and 2*Z*_load_ at the OBO level and saturation level, respectively. Then, the calculated *Z*_A2_ and *θ*_A_ are expressed as

(22) $$Z_{A2}=\sqrt{X^{2}+1+XX_{A}}$$

(23) $$\theta_{A}=arctan\left(\frac{2\sqrt{X^{2}+1+XX_{A}}}{X_{A}}\right)$$

The corresponding parameter values are summarized in Table 2. We can use these parameters in Table 2 to design the DPA working in the normalized frequency range of 0.4–1.

## 3. Design of The Proposed DPA

In this section, we present the complete design process for the enhanced bandwidth DPA based on the proposed load modulation network. In the previous section, the normalized frequency is used for analysis, and the normalized frequency band obtained is 0.4–1.0. Therefore, we can choose a different reference frequency in order to obtain actual working frequencies. In this paper, the reference frequency of 2.5 GHz is taken so that the actual working frequency range of 1–2.5 GHz can be achieved. This work focuses on the symmetrical DPA, so two CGH40010F transistors are employed to design the main PA and the auxiliary PA. The parameters of the transistors used can be obtained from the datasheet provided by the manufacturer. The designed circuits are based on Rogers R4350B substrate. The drain bias voltages of the main PA and auxiliary PA are both 28 V. The gate bias voltage of the main PA is −2.7 V, and that of the auxiliary PA is −5.8 V. For the selected transistor, the optimum load impedance *R*_OPT_ is 32 Ω when considering *V*_knee_.

### 3.1. Output Networks

**Figure 10:**
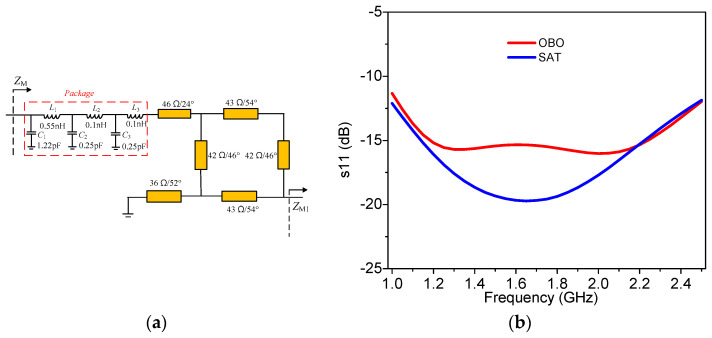
Main PA branch: (**a**) output network of the main PA; (**b**) simulated s11.

According to the description in Section 2, the output networks can be designed. Firstly, the output network of the main PA is designed based on the topology shown in Figure 7, where the phase *θ*_M_ varies from 46.7° to 0° in the frequency range of 1.0–2.5 GHz. In practical design, the package parameters of the transistor are considered [31] as part of the whole output network. Therefore, the injected transmission line and the modified coupler must adjust a little to meet the impedance requirements. The synthesized output circuit of the main PA is shown in Figure 10a. Figure 10b displays the simulated s11 of the designed output network under the condition of *Z*_M1,OBO_ and *Z*_M1,SAT_. It can be seen that the simulated S_11_ is smaller than −10 dB across the frequency range of 1–2.5 GHz at both saturation and OBO levels, which validates that the designed output network is effective at realizing the impedance conversion well at different power levels within the target frequency band.

**Figure 11:**
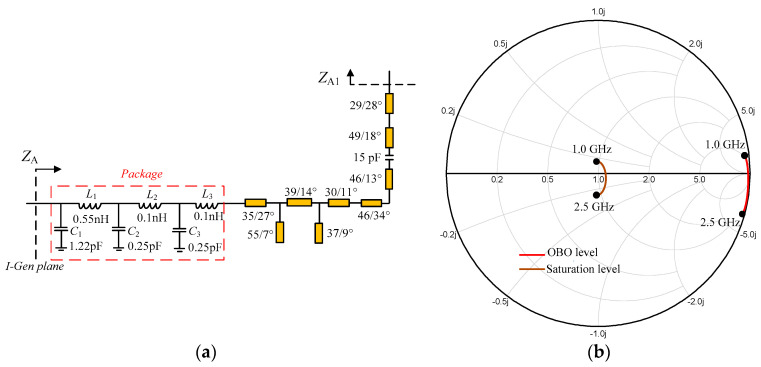
Auxiliary PA branch: (**a**) designed output network; (**b**) simulated *Z*_A_.

Next, the output network of auxiliary PA is designed by using the generalized transmission line obtained in Section 2. As per the analysis in Section 2, the characteristic impedance is changing from 1.52*R*_OPT_ to *R*_OPT_ over the target frequency range of 1–2.5 GHz, with the phase *θ*_A_ being between 85.4° and 90°. Similarly, the package parameters of the transistor are also included in the practical design. The complete output circuit including the package parameters is shown in Figure 11a. The simulated impedance *Z*_A_ is displayed in Figure 11b. It can be seen that the simulated *Z*_A_ is close to the open circuit at the OBO level and *R*_OPT_ at the saturation level is matched, in 1.0–2.5 GHz. These realized impedance trajectories indicate the effectiveness of the output circuit of the auxiliary PA branch.

### 3.2. Post-Matching Network

**Figure 12:**
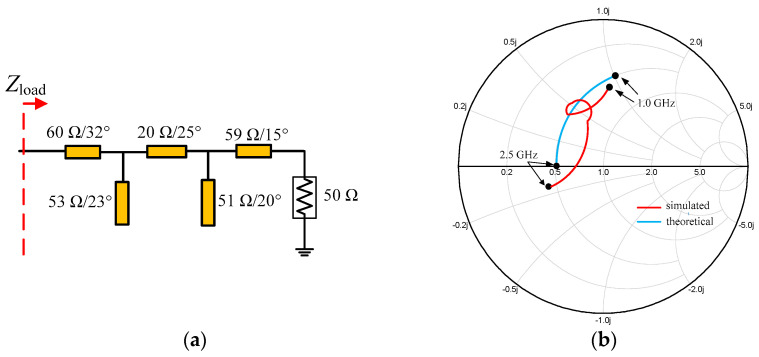
(**a**) Post-matching network; (**b**) simulated impedance *Z*_load_ (normalized *R*_OPT_).

In the previous analysis, the load impedance *Z*_load_ is a complex impedance that is equal to 0.5*R*_OPT_(1 + *jX*). The value of *X* changes from 1.37 to 0 in the frequency range of 1.0–2.5 GHz. Therefore, a post-matching network is needed to transform this load impedance to 50 ohms. The complete matching network is synthesized as shown in Figure 12a,b; the simulated *Z*_load_ is acceptable compared with the theoretical value of *Z*_load_.

### 3.3. Input Networks and Complete DPA

**Figure 13:**
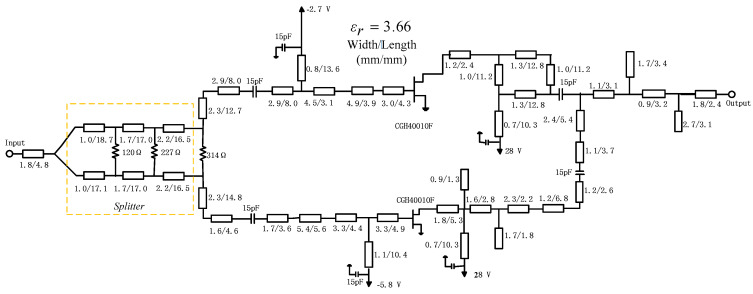
Completed DPA circuit with distributed parameters.

Firstly, an equal power divider is added to equally split the signal to the main PA and auxiliary PA. Here three stages are employed to meet bandwidth requirements. The input matching network is synthesized. Offset lines are also injected so that the signals of the two PA branches are combined in phase and transmitted to the load. Figure 13 shows the complete DPA with distributed parameters.

**Figure 14:**
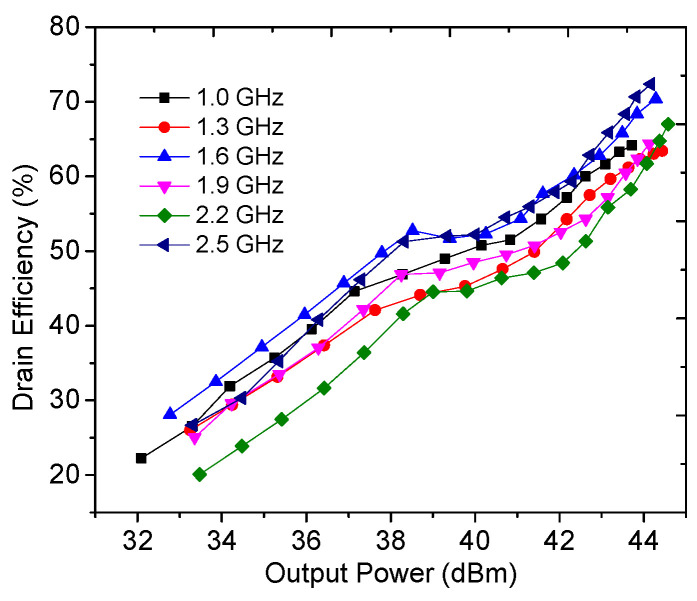
Simulated drain efficiency versus output power.

Figure 14 displays the simulated drain efficiency versus output power at several representative frequencies. As seen in Figure 14, the designed DPA can obtain over 43% drain efficiency at the 6 dB OBO level in the 1.0–2.5 GHz frequency range. At the same time, drain efficiency of over 62% can be obtained at the saturation level.

**Figure 15:**
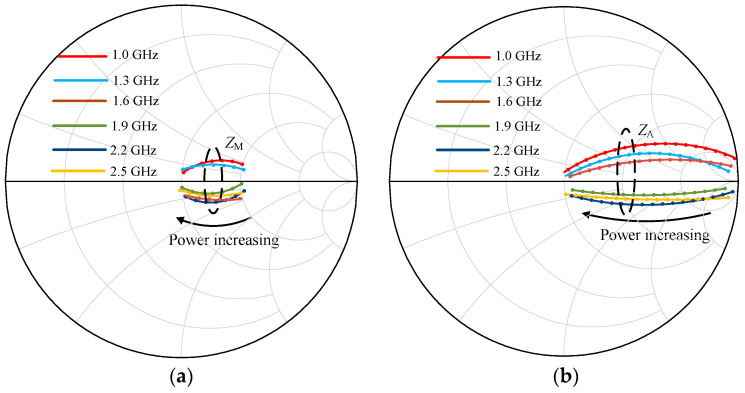
Trajectories of impedance Z_M_ and Z_A_ at several representative frequencies: (**a**) impedance Z_M_; (**b**) impedance Z_A_.

The impedance traces of *Z*_M_ and *Z*_A_ varying with power levels are plotted in Figure 15a,b at several representative frequencies. The load trajectories of *Z*_M_ and *Z*_A_ reveal that the designed DPA realizes the load modulation desired requirements across 1.0–2.5 GHz by using the proposed LMN structure.

## 4. Experiment and Results Analysis

**Figure 16:**
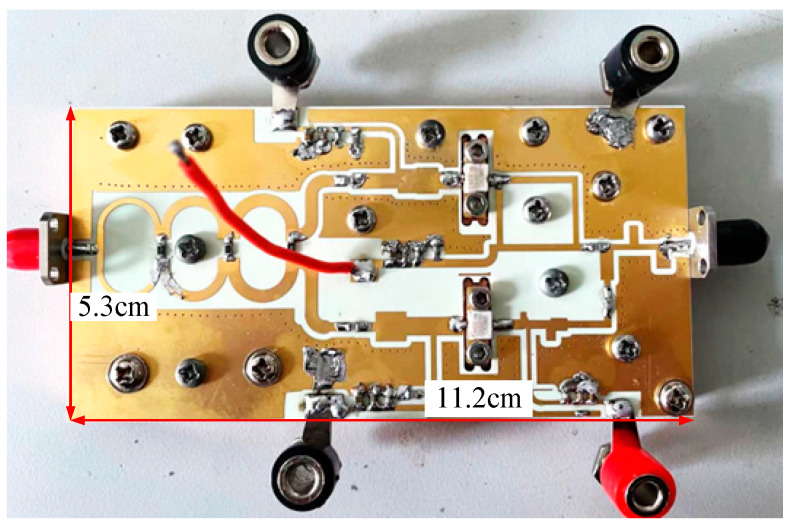
Photograph of the designed DPA.

A DPA circuit was fabricated using Rogers 4350B (*ε*_r_ = 3.66, *H* = 20 mils) substrate based on the schematic shown in Figure 13. Figure 16 shows a photograph of the fabricated DPA. The overall size of the circuit is 11.2 cm × 5.3 cm.

**Figure 17:**
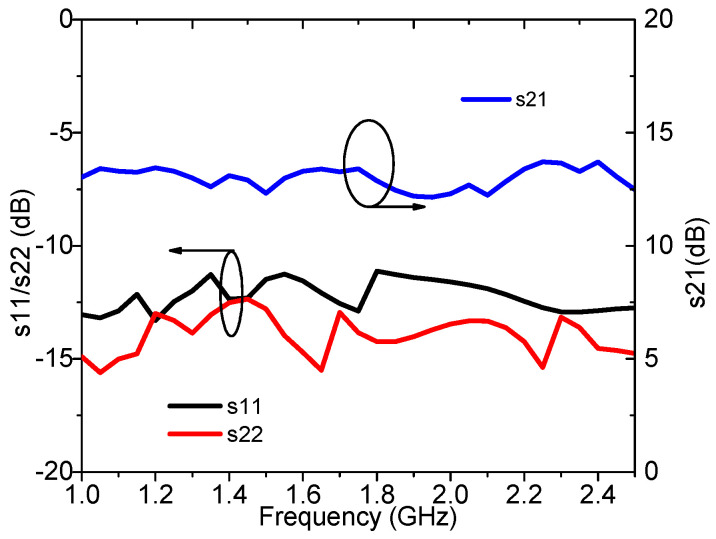
Measured s11, s22, and s21 of the designed DPA.

The measured S-parameters are plotted in Figure 17. In the frequency range of 1.0–2.5 GHz, the measured s21 is 12.2–13.6 dB, and the measured s11 and s22 are smaller than −10 dB.

### 4.1. Continuous Wave Testing

**Figure 18:**
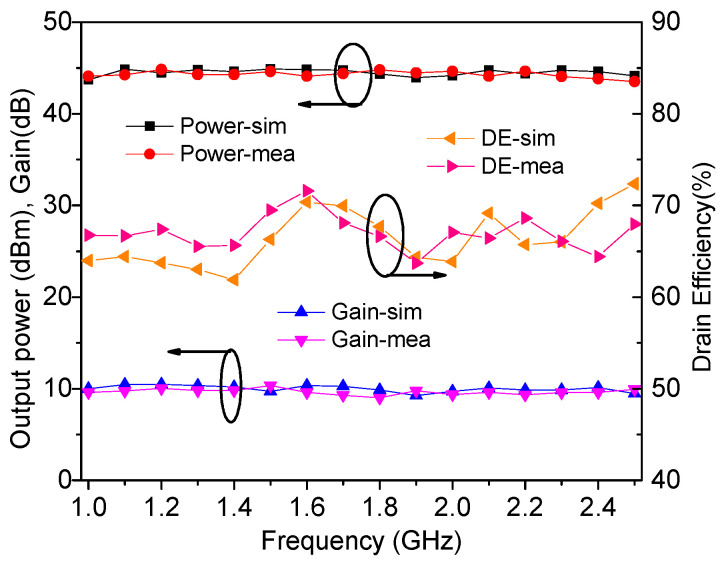
Simulated and measured performance at the saturation level.

**Figure 19:**
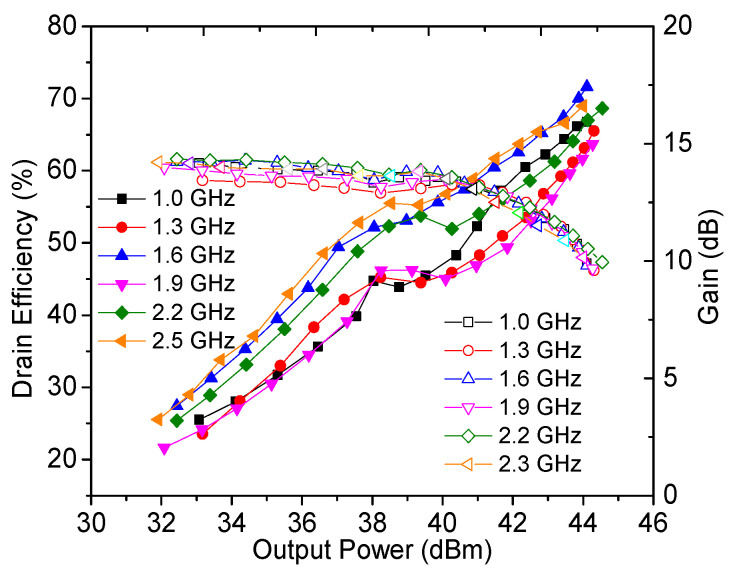
Measured drain efficiency and gain versus output power.

**Figure 20:**
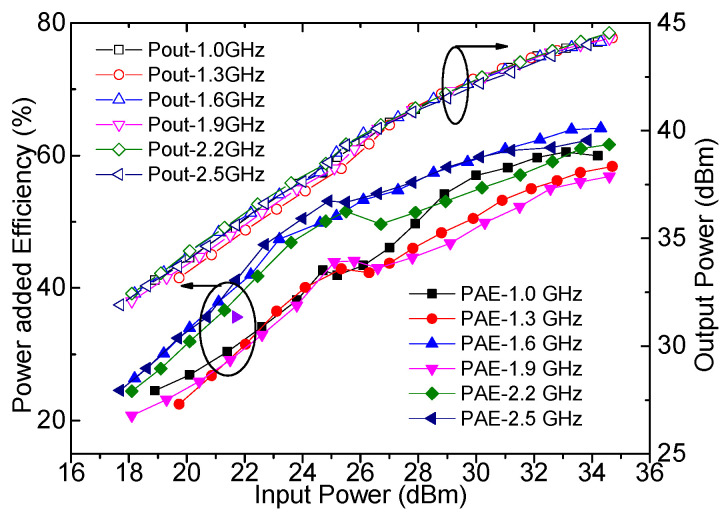
Measured power-added efficiency and output power versus input power.

The output power, drain efficiency, gain, and power-added efficiency were measured by using continuous wave signals and are plotted in Figure 18, Figure 19 and Figure 20. It can be seen from Figure 18 that an output power of 43.9–44.5 dBm with a drain efficiency of 63.7–71.6% can be obtained at the saturation level in 1.0–2.5 GHz, while the gain is between 9.6 dB and 10.3 dB. At the 6 dB OBO level, the realized drain efficiency is 45.2–53.7%. As shown in Figure 20, the power-added efficiency is between 42.3% and 64.1% within the dynamic load modulation range.

Table 3 lists several related and latest DPAs for comparison. It is obvious that the relative bandwidth of the proposed DPA is wider than that of the others except for that presented in [32], and the proposed DPA realizes similar performance, including in power, drain efficiency, and gain, when compared with the others.

### 4.2. LTE Testing at 40 MHz and 6.5 dB

**Figure 21:**
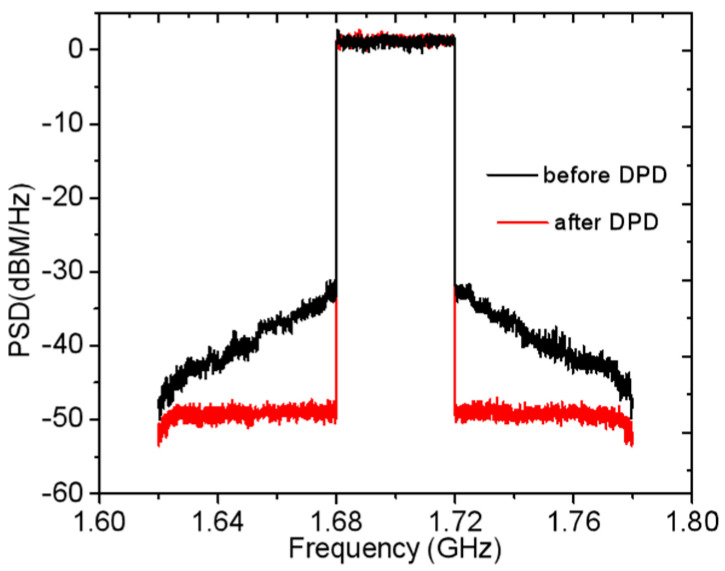
Measured ACLR at 1.7 GHz.

To characterize the linearity of the designed DPA, the adjacent channel ratio (ACLR) was measured by employing an LTE signal with a bandwidth of 40 MHz and PAPR of 6.5 dB. The measured ACLR was obtained when an average output power of 37.6 dBm was produced, as displayed in Figure 21. As shown in Figure 21, the ACLR value is less than −30.2 dBc at 1.7 GHz, which is better than −49.5 dBc after adopting digital predistortion (DPD).

## 5. Conclusions

This paper proposes a novel load modulation network consisting of two transmission lines and a modified coupler. The utilization of this modified coupler makes the load modulation network work well in a lower normalized frequency band, resulting in a larger relative bandwidth compared with previously used networks. The corresponding parameters at each operation frequency are solved to realize DPAs with a relative bandwidth of 85.6%. For validation, a broadband DPA operating in 1.0–2.5 GHz was implemented based on the obtained parameter solutions. Measurements reveal that the implemented DPA can deliver an output power of over 43.9 dBm with drain efficiency above 63% at the saturation level in the frequency range of 1.0–2.5 GHz. The drain efficiency is above 45.3% at the 6 dB OBO level across the same frequency band. Compared with the previous works, this work is close to the state-of-the-art relative bandwidth performance.

## Figures and Tables

**Table 1:** The values of *X* and *X*_A_ at representative frequencies.

Value	*X*	*X*_A1_	*X*_A2_
*f =* 0.4	1.37	−0.50	−3.37
*f =* 0.5	1	−0.40	−2.62
*f =* 0.6	0.72	−0.34	−2.15
*f =* 0.7	0.51	−0.30	−1.93
*f =* 0.8	0.32	−0.25	−2.09
*f =* 0.9	0.16	−0.15	−3.40
*f =* 1.0	0	0	∞

*X*_A1_ refers to the first solution of *X*_A_; *X*_A2_ refers to the second solution of *X*_A_.

**Table 2:** The values of the corresponding parameters.

Parameter	*f* = 0.4	*f* = 0.5	*f* = 0.6	*f* = 0.7	*f* = 0.8	*f* = 0.9	*f* = 1.0
*X*	1.37	1	0.72	0.51	0.32	0.15	0
*θ*_M_ (°)	46.7	37.4	30.1	23.7	17.0	8.9	0
*X*_A_	−0.42	−0.38	−0.35	−0.33	−0.26	−0.15	0
*Z*_A2_	1.52	1.27	1.12	1.04	1.00	1.00	1.00
*θ*_A_ (°)	85.9	85.7	85.4	85.5	86.2	87.6	90

**Table 3:** Several related and latest broadband DPAs.

Ref.	Freq (B.W.) (GHz)	Pout@SAT (dBm)	DE@SAT (%)	DE@ 6 dB OBO (%)	Device
[17]	1.1–2.4 (74%)	43.3–45.4	55–68	43.8–54.9	2 × 13 W GaN
[22]	1.6–2.7 (51%)	43.8–45.2	56–75.3	46.5–63.5	2 × 13 W GaN
[23]	3.3–3.75 (13%)	48–48.5	58–71	47–59	2 × 16 W GaN
[24]	1.2–2.8 (78%)	43.7–44.1	60.5–74.2	48.1–57.6	2 × 13 W GaN
[27]	1.0–2.5 (83%)	40–42	45–83	35–58	2 × 8 W GaN
[29]	2.8–3.55 (24%)	43–45	66–78	42–53	2 × 13 W GaN
[32]	1.5–3.8 (87%)	42.3–43.4	42–63	33–55	2 × 13 W GaN
[33]	1.2–2.4 (67%)	42–45	41.6–81	35–63	2 × 13 W GaN
[34]	1.4–2.55 (58%)	41.9–42.2	62–74	48–58	2 × 8 W GaN
**This work**	**1.0–2.5 (85.6%)**	**43.9–44.5**	**63.7–71.6**	**45.2–53.7**	**2 × 13 W GaN**

## Data Availability

Data are available on request from the authors.
